# Cost-Effectiveness of Mass Treatment with Azithromycin for Reducing Child Mortality in Malawi: Secondary Analysis from the MORDOR Trial

**DOI:** 10.4269/ajtmh.19-0622

**Published:** 2020-04-27

**Authors:** John D. Hart, Khumbo Kalua, Jeremy D. Keenan, Thomas M. Lietman, Robin L. Bailey

**Affiliations:** 1 London School of Hygiene and Tropical Medicine, London, United Kingdom;; ^2^Blantyre Institute for Community Outreach, Blantyre, Malawi;; ^3^Francis I Proctor Foundation and Department of Ophthalmology, University of California, San Francisco, San Francisco, Califorina

## Abstract

The recent Macrolides Oraux pour Réduire les Décès avec un Oeil sur la Résistance (MORDOR) trial reported a reduction in child mortality following biannual azithromycin mass drug administration (MDA). Here, we investigate the financial costs and cost-effectiveness from the health provider perspective of azithromycin MDA at the MORDOR-Malawi study site. During MORDOR, a cluster-randomized trial involving biannual azithromycin MDA or placebo to children aged 1–59 months, fieldwork-related costs were collected, including personnel, transport, consumables, overheads, training, and supervision. Mortality rates in azithromycin- and placebo-treated clusters were calculated overall and for the five health zones of Mangochi district. These were used to estimate the number needed to treat to avert one death and the costs per death and disability-adjusted life year (DALY) averted. The cost per dose of MDA was $0.74 overall, varying between $0.63 and $0.94 in the five zones. Overall, the number needed to treat to avert one death was 1,213 children; the cost per death averted was $898.47, and the cost per DALY averted was $9.98. In the three zones where mortality was lower in azithromycin-treated clusters, the number needed to treat to avert one death, cost per death averted, and cost per DALY averted, respectively, were as follows: 3,070, $2,899.24, and $32.31 in Monkey Bay zone; 1,530, $1,214.42, and $13.49 in Chilipa zone; and 344, $217.98, and $2.42 in Namwera zone. This study is a preliminary cost-effectiveness analysis that indicates azithromycin MDA for reducing child mortality has the potential to be highly cost-effective in some settings in Malawi, but the reasons for geographical variation in effectiveness require further investigation.

## INTRODUCTION

Azithromycin mass drug administration (MDA) is a key part of the global campaign to eliminate blinding trachoma.^1^ Research alongside trachoma programs has indicated a beneficial effect of azithromycin MDA on child morbidity indicators,^2–6^ and increasing evidence suggests this may include a reduction in child mortality.^7,8^

Despite accelerating reductions in child mortality over the past two decades, progress is not uniform, with more than one in 10 children still dying before their fifth birthday in six countries, five of them in sub-Saharan Africa.^9^ At current annual rates of reduction in child mortality, many countries will not meet the Sustainable Development Goals for reduction in child mortality by 2030, with the 2015 Millennium Development Goal for child mortality not likely to be met globally before 2026.^9^ Socioeconomic progress and improvements in health systems are key to long-term progress in reducing child mortality. However, in the short term, practical, cost-effective interventions to reduce child mortality are of particular interest.

The MORDOR trial, investigating the effect of biannual azithromycin MDA to children aged 1–59 months on child mortality in Malawi, Niger, and Tanzania, reported 14% lower mortality in azithromycin- compared to placebo-treated communities.^8^ The effect was not uniform, with mortality reduction of 6% in Malawi, 18% in Niger, and 3% in Tanzania. In addition, the effect differed significantly by age-group. Such heterogeneity raises important questions about who should be targeted and where, if wider country campaigns of azithromycin MDA to reduce child mortality are to be considered. Cost-effectiveness will play a key role in such policy decisions. As cost data were not collected uniformly at the three MORDOR study sites, this study reports child mortality in different geographical areas of the MORDOR trial in Malawi and the associated financial costs and cost-effectiveness of the intervention from the health provider perspective.

## METHODS

### MORDOR trial.

The MORDOR trial in Malawi was conducted in Mangochi district, one of the poorer districts in Malawi, with associated high birth rates, low literacy, and little formal employment.^10^ Mangochi district is shown on the map (Figure 2). The MORDOR trial methodology has been reported elsewhere.^8^ Briefly, MORDOR used a cluster-randomized, placebo-controlled trial design to assess the effects of biannual single-dose azithromycin MDA on mortality in children aged 1–59 months.

Clusters were defined as the catchment areas of health surveillance assistants (HSAs), which have approximately a total population of 1,000. The study included house-to-house visits for a total of four treatment rounds and five census visits between March 2015 and June 2017. The study cohort was updated biannually, as each census identified deaths as well as new births and migrations into or out of the study area. The MORDOR trial included 334 clusters that were randomly assigned to either the main mortality study (304 clusters) or for the assessment of morbidity outcomes (30 clusters). All clusters met the same inclusion criteria and were identified from a pre-baseline census. The cost-effectiveness analyses include all 334 clusters. Mangochi district is divided into five zones for health administration purposes. MORDOR fieldwork and the collection of cost data were organized by the five distinct geographical zones (Mangochi, Namwera, Chilipa, Monkey Bay, and Makanjira).

### Intervention.

Treatment was administered as 20 mg/kg azithromycin syrup or an equivalent volume of placebo. Young children were weighed to determine dose, and those old enough to stand were measured and given an approximate dose based on their height. Mass drug administration was performed by HSAs in their own communities with assistance from village volunteers and MORDOR field-workers, who also recorded the data using tablet devices. Adverse event reporting was encouraged for events occurring within 7 days of the MDA via the HSA to the study team.

### Randomization and blinding.

The study drug manufacturer (Pfizer Inc., New York, NY) labeled medicine bottles with eight letters corresponding to azithromycin and eight to placebo to reduce risks of unblinding. Study clusters were randomly assigned to a drug letter by the study statistician using the statistical package R (the R Foundation for Statistical Computing, Vienna, Austria). All field and laboratory staff, supervisors, data managers, and participants were blind to the treatment code until after all fieldwork was complete.

### Outcomes.

The primary prespecified outcome for this study was the cost-effectiveness of azithromycin MDA at the MORDOR-Malawi study site. The secondary outcome was a comparison of the differences in cost-effectiveness of azithromycin MDA by health zone in Mangochi district. The study was designed to assess cost-effectiveness from the perspective of service providers and not from a societal perspective.

### Cost data collection.

Cost data were collected at the 12-month follow-up visit and included personnel, transport, consumables, overheads, training, and supervision. Details of sources of cost data are provided in Table 1. Efforts were made to exclude costs related solely to research, such as the electronic capture of census and treatment data. All daily fieldwork costs were recorded and cross-checked with budgets and funds released by the MORDOR trial finance officer in coordination with the District Environmental Health Officer. Costs were collected in Malawian Kwacha (MWK) and converted to U.S. dollars ($) at the exchange rate of MWK700 = $1.00, the commonly used exchange rate in Malawi in 2016, where U.S. dollars are often used for major purchases. The value of the Malawian kwacha fluctuates significantly on the international market, and the exact exchange rate fluctuated above and below the value used for this study during the 12-month follow-up visit for MORDOR fieldwork between March and June 2016. All costs presented in this study are in 2016 USD. There were no missing cost data as costs were proactively captured during the fieldwork from budgets and receipts.

**Table 1:** Sources of cost data

Costs	How data were collected/costs estimated*
Training	
Environmental health officers’ (EHOs) phone credit, fuel, and allowance	Distributed to EHOs to attend training and make fieldwork supervisory visits (between $34.29 and $142.86 for each of the five zones depending on size, accessibility, and number of EHOs)
Health surveillance assistants’ (HSAs) allowance	As provided to each HSA ($17.86 each)
HSAs’ transport	Provided to HSAs traveling long distances to attend training ($2.86 per HSA)
Refreshments	Actual expenditure for training (between $34.29 and $100.00)
Hall hire (health center or teacher training hall)	Actual expenditure (between $8.57 and $10.00 per day)
Personnel	
HSAs’ per diem	Paid as agreed with the district ddministration ($3.57 per day for 5 days for a total of $17.86 per HSA); for the sensitivity analysis, each HSA salary was included for the allocated 1 week of work ($14.29 per HSA)
Volunteers’ per diem	Paid as agreed with the district ddministration ($2.14 per day for 5 days for a total of $10.71 per volunteer in each community)
MORDOR field-workers’ pay/allowances	Local enumerators staying at home paid $2.86 per day for 5 days for a total of $14.29 per community. Enumerators staying away from home paid $5.00 per day including per diem.
Field-worker accommodation	Actual expenditure for field-workers not able to return home at night (rooms costing between $2.86 and $6.43 per night)
Transport	
Vehicle depreciation	2 Toyota LandCruiser vehicles costing $60,000 each, with life expectancy of 10 years, assuming 345 working days per year ($17.39 per day per vehicle)
Fuel	Actual expenditure for fieldwork (between $1,721.56 and $2,752.24 per zone)
Boat fare	Actual expenditure for fieldwork (between $0.00 and $28.57 per zone, required to get to some remote communities without road access)
Bicycle fare	Actual expenditure for fieldwork ($1.43 per day for 3 days for one HSA to reach remote community)
Consumables	
Study drug	No cost for donated drug; for the sensitivity analysis, cost of azithromycin was taken from the WHO International Product Price Guide for 2015 ($0.94 per 30 mL bottle of syrup, with three doses per bottle, as allocated for fieldwork)
Waste bags	Actual expenditure for fieldwork ($28.57 per zone)
Overheads	
Office and storage space rent	Rental cost for two offices and storage space during the fieldwork ($1,128.57/month)
Office security and upkeep	Security and upkeep costs for rented premises during the fieldwork ($102.86/month)
Office utilities	Utilities for rented premises during the fieldwork ($205.71/month)
Supervision	
MORDOR staff per diems, accommodation, and lunch allowances	2–3 staff plus drivers ($14.29 per diem when staying away from Mangochi town; rooms costing between $4.29 and $8.57 per night; and lunch allowance of $3.57 provided every day)

* All costs were measured during the 12-month follow-up round of MORDOR activities.

Personnel costs included per diems for HSAs, village volunteers, drivers, MORDOR field-workers, and supervisors. Salary costs were not included for the main analysis, although HSA salaries were included in a subsequent sensitivity analysis. Training costs were included for HSAs to conduct the MDA; separate training costs for field-workers specifically on the use of tablet devices were excluded. Morbidity assessment costs for field-workers collecting samples and laboratory staff working on research activities were excluded.

Vehicle depreciation was calculated daily for two MORDOR vehicles by dividing the capital cost of the new vehicles by their 10-year life expectancy multiplied by 345, an assumed number of working days per year. Fuel costs were taken from the fuel receipts used specifically for the fieldwork in each zone. Additional transport costs, including bicycle and boat taxis, were included as required.

Azithromycin and placebo suspension were donated by Pfizer, so no costs were included for study drug in the main analysis, although the value of azithromycin was included in a sensitivity analysis. Overheads included rental costs for office and storage space in two locations and associated security, utilities, and upkeep costs. Supervision costs were mainly related to the MORDOR trial supervisors; zonal environmental health officer’s (EHO’s) costs were included to attend trainings.

### Cost-effectiveness calculations.

Total costs were summed for each of the five zones of Mangochi district. The cost per treatment in each zone and overall was calculated using the total number of treatments distributed at the 12-month follow-up visit. Mortality rates per 1,000 person-years were calculated for azithromycin- and placebo-treated clusters using the total deaths and person-years of follow-up over the full 2 years and four intercensal periods of the MORDOR trial. The number needed to treat to avert one death was calculated from the rate difference between azithromycin and placebo clusters. The cost per death averted was then calculated by zone and overall using the number needed to treat and cost per treatment. Cost per disability-adjusted life year (DALY) averted was also calculated from years of life lost in the study using the mean age of mortality of children in the study area and WHO standard life tables with no age weighting or time discounting.^11,12^

A lifetime time horizon was used for this study to capture all effects associated with a round of azithromycin MDA, as opposed to assuming, in effect, that for lives saved during the intervention, those individuals would die instantly at the end of any shorter follow-up period. The evidence available to date regarding the effect of azithromycin MDA on child mortality does not indicate any reduction in effect with subsequent rounds of treatment. Indeed, the aggregate efficacy of azithromycin as compared with placebo tended to increase with each progressive round of treatment in the MORDOR trial.^8^ In addition, most of the protective effect of azithromycin MDA occurred in the 3 months after distribution, so this study assumed that each biannual MDA has an equal and independent effect on mortality.^13^

Cost-effectiveness of the intervention was compared with the WHO willingness-to-pay thresholds, specifically the estimate that an intervention costing less than three times the national annual GDP per capita per DALY avoided, may be considered cost-effective, whereas one costing less than the national annual GDP per capita may be considered highly cost-effective.^14^ The WHO willingness-to-pay criteria applied to Malawi in 2016, with GDP per capita of $316, indicate that a cost-effective intervention would cost less than $948 per DALY averted and a highly cost-effective intervention would cost less than $316 per DALY averted.^15^

Sensitivity analyses were conducted: firstly, adding the value of azithromycin using the median price estimated for the buyer using the WHO International Product Price Guide and, secondly, adding both the value of azithromycin and the salaried time of HSAs.^16^

### Ethical approval.

Approval for the MORDOR trial was obtained from the College of Medicine, University of Malawi; the London School of Hygiene and Tropical Medicine; and the UCSF Committee on Human Research. Oral informed consent was obtained from the guardians of participants. There were no incentives for participation.

## RESULTS

The baseline characteristics of clusters and study participants were similar between placebo and azithromycin arms at baseline, as shown in Table 2. The costs for activities related to the MDA varied by zone and overall and are listed in Table 3. The least costly zone for MDA was Namwera, costing $0.63 per dose distributed, slightly cheaper than Mangochi zone at $0.66. The most expensive zone was Monkey Bay, costing $0.94 per dose distributed. The mean cost per dose administered overall zones was $0.74.

**Table 2:** Baseline characteristics of study clusters and participants

	Placebo	Azithromycin
Number of clusters	167	167
Number of children enrolled	42,825	43,105
Number of children per cluster	286 (SD: 133)	285 (SD: 127)
Gender, male (%)	50.0	50.0
Age-group (%)		
1–11 months	18.7	18.6
12–59 months	81.3	81.4

**Table 3:** Itemized costs for MDA by zone

	Cost (USD)					
	Monkey bay	Chilipa	Makanjira	Namwera	Mangochi	Total
Training						
Environmental health officers (phone credit, fuel, and allowance)	34.29	34.29	114.29	85.71	142.86	411.43
Health surveillance assistants’ (HSAs) allowance	263.57	771.43	263.57	457.14	708.57	2,464.28
HSAs’ transport	57.14	42.86	28.57	57.14	42.86	228.57
Refreshments	34.29	54.29	43.57	67.14	100.00	299.29
Hall hire (health center or teacher training hall)	8.57	17.14	17.14	17.14	20.00	80.00
Personnel						
HSAs’ per diem	732.14	964.29	732.14	1,535.71	2,214.29	6,178.57
Volunteers’ per diem	439.29	578.57	439.29	921.43	1,328.57	3,707.14
MORDOR field-workers’ pay plus allowances	1,121.43	1,191.43	1,178.57	1,755.71	1,645.71	6,892.86
Field-worker accommodation	650.00	1,041.43	640.71	900.00	855.00	4,087.14
Transport						
Vehicle depreciation	660.87	660.87	660.87	904.35	904.35	3,791.33
Fuel	2,421.87	2,305.27	2,043.78	1,721.56	2,752.24	11,244.73
Boat fare	22.86	0.00	28.57	0.00	0.00	51.43
Bicycle fare	4.29	0.00	0.00	0.00	0.00	4.29
Consumables						
Study drug	0.00	0.00	0.00	0.00	0.00	0.00
Waste bags	28.57	28.57	28.57	28.57	28.57	142.86
Overheads						
Office and storage space rent	846.43	846.43	846.43	1,128.57	1,128.57	4,796.43
Office security and upkeep	77.14	77.14	77.14	102.86	102.86	437.14
Office utilities	154.29	154.29	154.29	205.71	205.71	874.29
Supervision						
MORDOR staff per diems, accommodation, and lunch allowances	1,228.57	257.14	930.00	1,796.43	542.86	4,755.00
Total cost	8,785.60	9,025.43	8,227.51	11,685.20	12,723.02	50,446.77
Number of children treated at the 12-month follow-up	9,304	11,369	9,794	18,451	19,197	68,115
Cost per treatment	0.94	0.79	0.84	0.63	0.66	0.74

**Figure 1.**
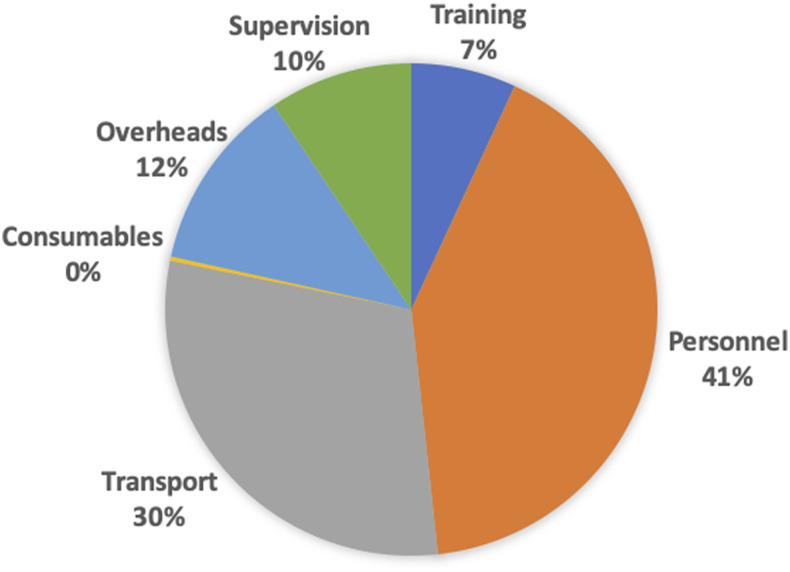
Breakdown of mass drug administration costs by broad category. This figure appears in color at www.ajtmh.org.

A breakdown of costs by broad category is shown in Figure 1. The highest proportion of costs (41%) was for personnel associated with conducting the MDA, followed by transport (30%), overheads (12%), supervision (10%), and training (7%).

**Figure 2.**
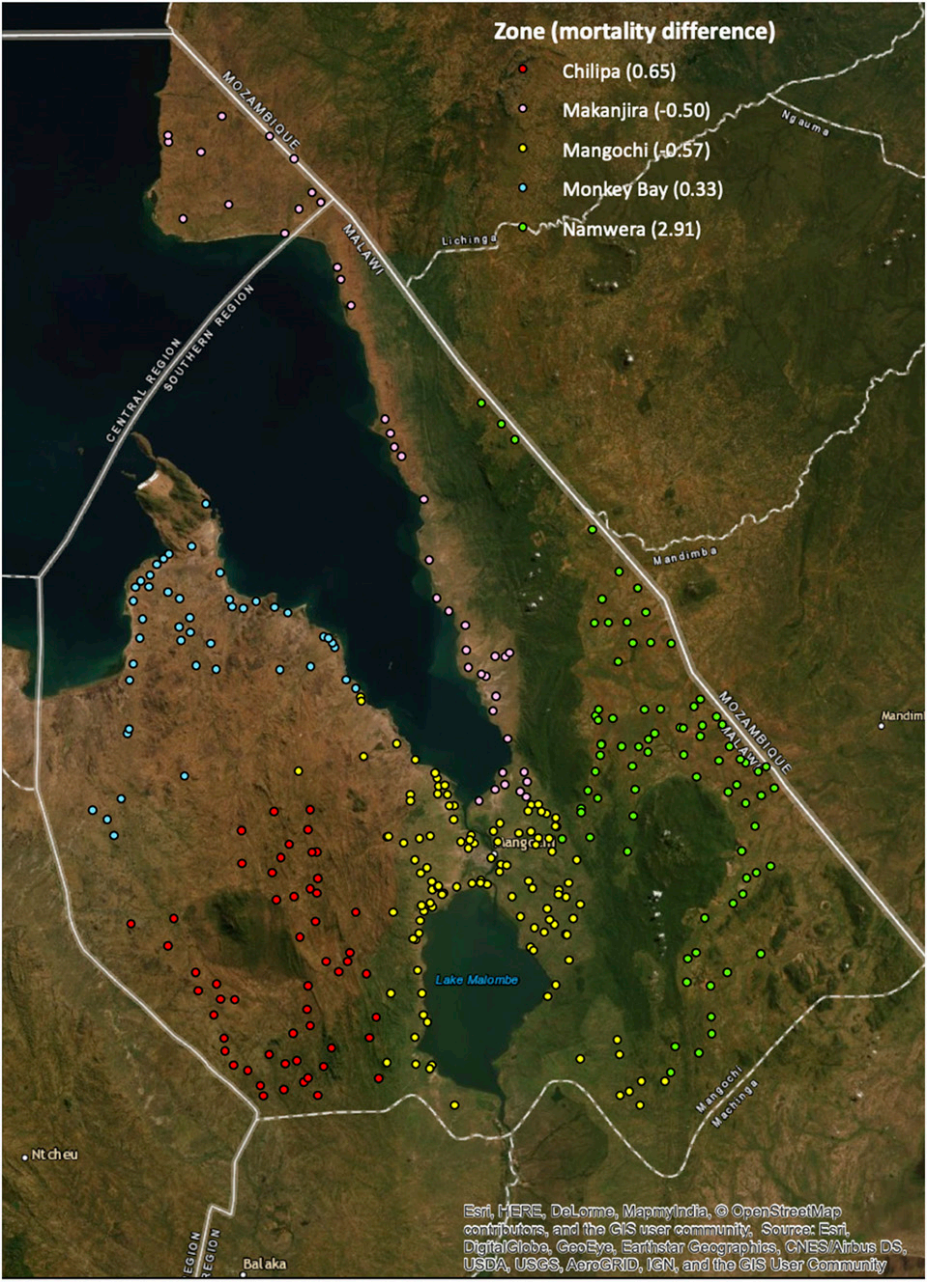
Mortality rate difference by zone between placebo- and azithromycin-treated clusters (deaths per 1,000 person-years); negative rate difference indicates higher mortality in azithromycin- than placebo-treated clusters.

The mortality rates by zone for placebo- and azithromycin-treated clusters and calculation of the rate difference between treatment arms are shown in Table 4. Study clusters are plotted in Figure 2, indicating the mortality rate difference in azithromycin and placebo arms by zone.

**Table 4:** Mortality rate by treatment arm and cost-effectiveness by zone

Zone	Monkey Bay	Chilipa	Makanjira	Namwera	Mangochi	Total
Cost/treatment (USD)	0.94	0.79	0.84	0.63	0.66	0.74
Person-years in placebo clusters (thousands)	7.58	12.28	10.08	18.05	18.94	66.93
Deaths in placebo clusters	53	134	89	191	153	620
Mortality rate in placebo clusters (deaths per 1,000 person-years, 95% CI)	6.99 (5.34–9.15)	10.91 (9.21–12.93)	8.83 (7.18–10.87)	10.58 (9.18–12.19)	8.08 (6.89–9.46)	9.26 (8.56–10.02)
Person-years in azithro clusters (thousands)	10.96	11.60	9.33	17.59	17.36	66.84
Deaths in azithro clusters	73	119	87	135	150	564
Mortality rate in azithro clusters (deaths per 1,000 person-years, 95% CI)	6.66 (5.30–8.38)	10.26 (8.57–12.28)	9.32 (7.56–11.50)	7.67 (6.48–9.08)	8.64 (7.36–10.14)	8.44 (7.77–9.16)
Rate difference (deaths per 1,000 person-years)	0.33	0.65	−0.50	2.91	−0.57	0.82
Number needed to treat to avert one death	3,070	1,530	–	344	–	1,213
Cost per death averted (USD)	2,899.24	1,214.42	–	217.98	–	898.47
Cost per DALY averted (USD)	32.21	13.49		2.42		9.98

Overall, the number needed to treat with azithromycin MDA to avert one death was 1,213 children, the cost per death averted was $898.47, and the cost per DALY averted was $9.98 (Table 4). The cost per DALY averted would indicate this intervention has the potential to be highly cost-effective according to the WHO willingness-to-pay criteria (less than the GDP per capita of $316). The greatest effect was in the Namwera zone, with the number needed to treat as 344, cost per death averted of $217.98, and cost per DALY averted of $2.42. In two zones, Makanjira and Mangochi, the mortality rate estimate was higher in azithromycin- than placebo-treated clusters, so a figure for cost per death averted could not be computed for these zones.

Sensitivity analyses are shown in Table 5. With the addition of the cost of azithromycin, the cost per death averted overall increased to $1,278.59 and the cost per DALY averted to $14.21. With the cost of HSA salaries for the duration of the work also included, the cost per death averted further increased to $1,366.63 and the cost per DALY averted to $15.18.

**Table 5:** Sensitivity analyses for cost-effectiveness including cost of azithromycin and HSAs’ salaries

Zone	Monkey Bay	Chilipa	Makanjira	Namwera	Mangochi	Total
Including the cost of azithromycin						
Cost/treatment (USD)	1.26	1.11	1.15	0.95	0.98	1.05
Cost per death averted (USD)	3,861.27	1,693.74	–	325.82	–	1,278.59
Cost per DALY averted (USD)	42.90	18.82	–	3.62	–	14.21
Including the cost of azithromycin and HSAs’ salaries						
Cost/treatment (USD)	1.32	1.18	1.21	1.01	1.07	1.13
Cost per death averted (USD)	4,054.55	1797.54	–	348.74	–	1,366.63
Cost per DALY averted (USD)	45.05	19.97	–	3.87	–	15.18

HSA = health surveillance assistant.

## DISCUSSION

This study estimated the cost-effectiveness of azithromycin MDA in the MORDOR trial in Malawi and compared cost-effectiveness by geographical zone in the intervention district. The cost per treatment distributed overall was $0.74, and the cost per death averted was $898.47. The cost per treatment delivered varied by up to 50% between zones. Cost-effectiveness varied considerably by zone; indeed, in two zones, the mortality rate was higher in azithromycin-treated than placebo-treated clusters. In the zones where mortality was lower in azithromycin-treated clusters, cost per death averted varied from $217.98 to $2,899.24.

The MORDOR trial was not powered to identify a mortality difference at any single site, but using the effect estimate of mortality and related cost-effectiveness, under $1,000 per death averted is comparable to recommended interventions for reducing child mortality, such as integrated management of childhood illness^17^ and seasonal malaria chemoprophylaxis.^18^

Azithromycin MDA has mostly been conducted by country programs for the control of trachoma, with the estimated costs varying considerably, from $0.50 or less per person treated^19–21^ to $1.50 or more.^22,23^ Other MDA programs in sub-Saharan Africa report generally similar costs: a systematic review of lymphatic filariasis MDA programs between 2000 and 2014 reported mean financial cost per treatment of $0.46 (adjusted to 2014 USD), mean economic cost excluding donated drug of $0.56, and economic cost including donated drug value of $1.32.^24^ Variation in MDA costs by country will be influenced considerably by the costs for staff involved, such as the use of volunteers or health workers. The costs for treatment of children only, as in MORDOR, as opposed to whole community MDA, would be expected to increase as more time will be required to identify eligible individuals. In addition, as fewer treatments are distributed when targeting children only, economies of scale are less, which may also increase the cost per treatment.

The largest component of the cost of the MDA was personnel costs, followed by transport. Personnel costs have been reported as the main driver of azithromycin MDA costs in other studies.^21,22^ One of these studies also found transport to contribute the second highest costs^22^, but the other study, in relatively accessible areas of the Gambia, found transport to not contribute a major share of costs.^21^ It is expected that these costs will vary considerably by geographical location and country.

The two main components of personnel costs were HSAs’ and MORDOR field-workers’ per diems. Health surveillance assistants collect a monthly salary, the costs of which were included in a sensitivity analysis, and receive per diems for any additional activities required of them beyond their few days rostered work at a nearby health center; this includes government campaigns, such as azithromycin MDA for trachoma, and interventions with other development partners. The per diems vary considerably; those recorded for this study were slightly higher than those paid by the government and significantly lower than those paid by other organizations.

MORDOR field-worker costs were included because of the key role these staff played in the context of the MORDOR trial supporting the HSAs with the MDA, although they were also tasked to update the census and record all treatments distributed. These exact costs would not be required for a government MDA program, although additional support with logistics would be needed from district and national health staff. The time spent during the MDA to complete census is also likely to have slowed the MDA fieldwork and increased costs. The level of supervision included in this analysis was believed to be similar to that which would be performed by EHOs for a government program; the MORDOR trial supervisors’ per diems and other support costs similar to the allowance and transport costs required for supervision during a government campaign.

Training costs accounted for 7% of the total MDA costs. These may be reduced slightly with repeated program distributions although some refresher training would usually be recommended. Capital costs for setting up the program were converted to ongoing running costs, for example, by including a daily depreciation cost for vehicles rather than front-loading the initial capital cost. This was performed to provide a simple estimate of the financial costs for a program to provide the intervention.

Interpretation of the findings of this study, providing the first estimates of cost-effectiveness from the MORDOR trial, must consider that it was designed to assess costs from the perspective of the health provider. Costs of donated drug were excluded, although a sensitivity analysis was performed, which indicated a potential increase in cost per treatment of 42% when the value of azithromycin was included and a 53% increase when both azithromycin and HSA salaries were included. Additional economic indicators associated with an analysis from the societal perspective, such as opportunity costs, have not been considered in this study.

The analysis in this study will be relevant to Malawi with its current level of health system and health programs in place, termed intervention mix constrained cost-effectiveness analysis.^25^ This evaluates the cost-effectiveness of additional interventions (in this case azithromycin MDA) with respect to the existing set of interventions, including interventions improving child mortality that may or may not have overlapping benefits with azithromycin MDA. Indeed, many health interventions are likely to interact with azithromycin MDA in terms of effect on child health, and therefore, in areas with improved health access and interventions, azithromycin is likely to provide lesser additional benefit.

To attempt to isolate the cost-effectiveness independent of other health interventions in a study area, termed generalized cost-effectiveness analysis, requires the analyst to consider the future effects if all health sector resources could be reallocated^25^ and is beyond the scope of this study. Generalizability of the effects of azithromycin MDA in Malawi as an intervention for child health to other settings may be possible to some extent depending on local child mortality rate, child health interventions, and the as-yet undefined mechanisms by which azithromycin MDA may reduce child mortality.

Future evaluations regarding the cost-effectiveness of azithromycin MDA for reducing child mortality should aim to estimate the cost-effectiveness when using the full economic cost. Further understanding of the mechanism of action is required, and subsequent analyses should assess the costs associated with targeting the intervention in settings where it will be effective. A further consideration is the potential for the development of macrolide resistance and lower effectiveness with continued biannual MDA as a possible consequence. Evidence from the trachoma field indicates that although resistance (in nontarget organisms) develops following azithromycin MDA, this usually decreases to relatively low levels 6 months posttreatment.^26,27^ The levels of resistance do vary considerably by country, however, and in Ethiopia, macrolide resistance in carried *Streptococcus pneumoniae* has been reported at 20–30% 12–24 months post-cessation of MDA.^28^

Evidence from the MORDOR trial in Niger indicates macrolide resistance in nasopharyngeal *S. pneumoniae* isolates 6 months after the fourth biannual MDA was higher in azithromycin-treated communities than placebo communities, although still at the relatively low community mean level of 12% macrolide resistance.^29^ Despite these low levels of resistance, the effect size appeared to increase over the four intercensal periods of the MORDOR trial, and indeed, with continued treatment for a third year of biannual MDA in the MORDOR II trial in Niger, effectiveness remained similar.^30^ This study assessed the cost-effectiveness per round of MDA over the first 2 years of the intervention in Malawi, although there is no evidence that the effect may decrease with subsequent distributions in this setting.

In conclusion, these initial findings from the MORDOR trial indicate that azithromycin MDA for reducing child mortality has the potential to be a highly cost-effective intervention in the Malawian setting, but that there is considerable variation by geographical location. A greater understanding of the reasons for geographical variation in effectiveness would be desirable before wider implementation of the intervention.
